# Cardiovascular adverse effects of antiviral therapies for COVID-19: Evidence and plausible mechanisms

**DOI:** 10.1038/s41401-024-01382-w

**Published:** 2024-09-09

**Authors:** Eileen Chen, Lei Xi

**Affiliations:** 1 Virginia Commonwealth University School of Medicine (M.D. Class 2027), Richmond, VA 23298 USA; 2 Pauley Heart Center, Division of Cardiology, Department of Internal Medicine, Virginia Commonwealth University, Richmond, VA 23298-0204 USA

**Keywords:** COVID-19, SARS-CoV virus, antiviral drugs, heart, adverse drug reaction

## Abstract

Antiviral therapeutics have made a critical contribution in mitigating the symptoms and clinical outcomes of the coronavirus disease of 2019 (COVID-19), in which a single-stranded RNA viral pathogen, the severe acute respiratory syndrome coronavirus 2 (SARS-CoV-2), causes multi-organ injuries. Several antivirals were widely prescribed to treat COVID-19, either through the emergency use authorization (EUA) by the governmental regulatory agencies (i.e., remdesivir, paxlovid, molnupiravir, and the SARS-CoV-2-targeted monoclonal antibodies - tixagevimab and cilgavimab), as well as the repurposed use of the existing antiviral or antimalarial drugs (e.g., hydroxychloroquine, chloroquine, and ivermectin). Despite their efficacy in ameliorating COVID-19 symptoms, some adverse side-effects of the antivirals were also reported during the COVID-19 pandemic. Our current review has aimed to gather and extrapolate the recently published information concerning cardiovascular adverse effects caused by each of the antivirals. We also provide further discussion on the potential cellular mechanisms underlying the cardiovascular adverse effects of the selected antiviral drugs, which should be carefully considered when evaluating risk factors in managing patients with COVID-19 or similar infectious diseases. It is foreseeable that future antiviral drug development assisted with the newest artificial intelligence platform may improve the accuracy to predict the structures of biomolecules of antivirals and therefore to mitigate their associated cardiovascular adversities.

## Introduction

**Fig. 1:**
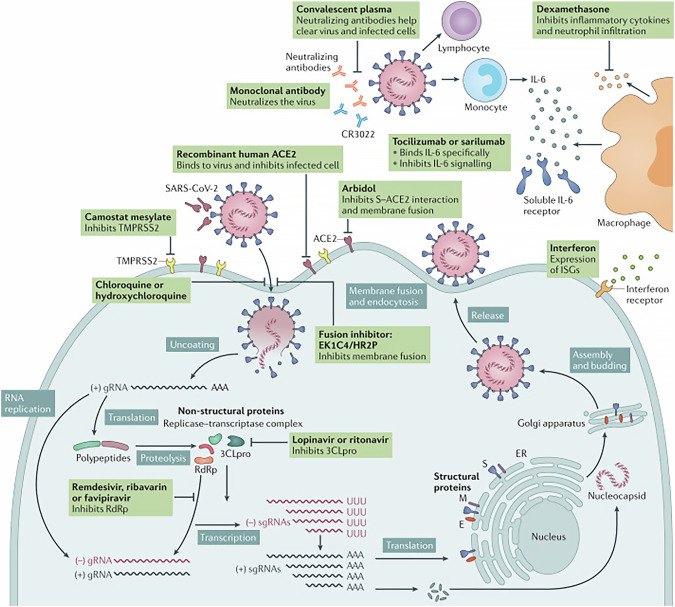
SARS-CoV-2 replication and potential therapeutic targets. Potential antivirals target the different steps of severe acute respiratory syndrome coronavirus 2 (SARS-CoV-2) replication, ranging from receptor binding, entry and fusion to replication. Furthermore, immunoglobulin-based and immunomodulatory drugs are potential therapeutics as well. Note that robust data on clinical efficacy are lacking for most of these treatments so far. *Abbreviations*: 3CLpro 3C-like protease, ACE2 angiotensin-converting enzyme 2, CR3022 a SARS-CoV-specific human monoclonal antibody, E envelope protein, EK1C4 lipopeptide derived from EK1 which is a pan-coronavirus fusion inhibitor targeting the HR1 domain of the spike protein, ER endoplasmic reticulum, gRNA genomic RNA, HR2P heptad repeat 2-derived peptides of SARS-CoV-2 spike protein, IL-6 interleukin-6, ISG interferon-stimulated gene, M membrane protein, RdRp RNA-dependent RNA polymerases, sgRNA subgenomic RNA, S spike protein, TMPRSS2 transmembrane protease serine protease 2.

Antivirals have played a vital role in the treatment of the coronavirus disease of 2019 (COVID-19) caused by severe acute respiratory syndrome coronavirus 2 (SARS-CoV-2) which is a single-stranded RNA virus. The genome of SARS-CoV-2 is located within a nucleocapsid formed from nucleocapsid protein which is packed within an envelope consisting of three proteins: membrane protein, spike protein, and envelope protein [1, 2]. The protein that guides coronavirus entry into host cells is mediated by the spike protein that is composed of two subunits: S1 and S2. The S1 subunit consists of an N-terminal domain (NTD) and a receptor-binding domain (RBD) and is responsible for binding to the receptor on the host cell. The S2 subunit consists of a fusion peptide, heptad repeat 1, central helix, connector domain, heptad repeat 2, transmembrane domain, and cytoplasmic tail. The S2 subunit is responsible for fusing the membranes of the virus and host cells [3–5]. The RBD of S1 uses angiotensin-converting enzyme 2 (ACE2) as the cell receptor. Receptor binding induces the dissociation of S1 with ACE2, prompting the S2 to transition from a pre-fusion state to a post-fusion stable state that is necessary for membrane fusion [6–10]. ACE2 is not only a SARS-CoV-2 receptor, but also acts to counterbalance the effects of ACE on the cardiovascular system. ACE converts angiotensin I to angiotensin II, which exhibits vasopressor and proinflammatory activities [11, 12]. Angiotensin II accumulation also causes a massive release of cytokines which triggers an uncontrolled immune response and tissue damage – the typical hallmarks of severe COVID-19 [13–15]. Therefore, it is conceivable that both SARS-CoV-2 infection and the SARS-CoV-2-targeted antivirals may have profound effects on the cardiovascular system. Under these contexts, the present review focuses on providing an updated analysis on the potential cardiovascular adverse effects of the commonly used antiviral therapeutics against COVID-19 during the past 3 to 4 years under the pandemic, as succinctly illustrated in Fig. 1.

## Studies on cardiovascular side-effects of antivirals prior to COVID-19 pandemic

Approximately six decades ago in 1963, the first antiviral drug, idoxuridine was approved for the treatment of herpesvirus-1 ocular infections in cats [16, 17]. Since then, many more antivirals have been developed for treating different infectious diseases with viral pathogens. The current armamentarium for the chemotherapy of viral infections consists of 37 licensed antiviral drugs [18]. For the treatment of human immunodeficiency virus (HIV) infections, the commonly used antivirals include (i) the nucleoside reverse transcriptase inhibitors (NRTIs) zidovudine, didanosine, zalcitabine, stavudine, lamivudine, abacavir and emtricitabine; (ii) the nucleotide reverse transcriptase inhibitors (NtRTIs) tenofovir, disoproxil, fumarate; (iii) the non-nucleoside reverse transcriptase inhibitors (NNRTIs) nevirapine, delavirdine and efavirenz; (iv) the protease inhibitors saquinavir, ritonavir, indinavir, nelfinavir, amprenavir, lopinavir (combined with ritonavir at a 4/1 ratio) and atazanavir; and the viral entry inhibitor enfuvirtide. For the treatment of chronic hepatitis B virus (HBV) infections, lamivudine as well as adefovir dipivoxil have been approved. Among the anti-herpesvirus agents, acyclovir, valaciclovir, penciclovir, famciclovir, idoxuridine, and trifluridine, as well as brivudin, are used in the treatment of herpes simplex virus (HSV) and/or varicella-zoster virus (VZV) infections; and ganciclovir, valganciclovir, foscarnet, cidofovir, and fomivirsen (have proven useful in the treatment of cytomegalovirus (CMV) infections in immunosuppressed patients. Following amantadine and rimantadine, the neuraminidase inhibitors zanamivir and oseltamivir have recently become available for the therapy of influenza virus infections. Ribavirin has been used in the treatment of respiratory syncytial virus (RSV) infections, and the combination of ribavirin with interferon-alpha has received increased acceptance for the treatment of hepatitis C virus (HCV) infections [18]. The duration of treatment varies from a few days (HSV, VZV, influenza virus infections) to several months or years (HIV, HBV, and HCV infections), depending on whether it is an acute/primary (i.e. influenza) or recurrent (i.e. HSV, VZV) infection or chronic, persistent (i.e. HIV, HBV, HCV) infection [18].

As summarized in Table 1, a study in 2013 investigated the cardio-metabolic effects of HIV protease inhibitors (Lopinavir/Ritonavir). Male Wistar rats received either: mock surgery (sham), vehicle-, or protease inhibitor-containing pump for a total of 8 weeks. The study revealed that protease inhibitor treatment increased serum LDL-cholesterol levels, triglyceride content in heart and liver tissues, and higher HDL-cholesterol levels. Further analyses of heart function at baseline revealed significantly decreased contractile force with protease inhibitor treatment [19]. In human endothelial cells, treatment with ritonavir resulted in significant endothelial cell injury, which was demonstrated by a decrease of cell viability and an increase in cytotoxicity. Since endothelial injury or dysfunction is a major cause of atherosclerotic lesion formation and progression, ritonavir-induced endothelial injury may contribute to its vascular complications [20]. Cardiovascular effects were also seen after treatment with interferon-α which is used for the treatment of many viral and neoplastic diseases. One study in 1991 evaluated 138 patients who were given interferon-α. Among these patients, six cardiac manifestations (4.3%) were observed, among which one unreported complete atrioventricular block. These side effects occurred at low doses in patients whose mean age was 63 years; 5 of the 6 patients had previous cardiovascular incidents. These findings advise prudence in high-risk patients [21].

**Table 1:** Representative studies on cardiovascular complications of the antivirals prior to the COVID-19 pandemic.

Study/Trial Information	Subject Characteristics	Key Clinical Findings	Publications
1. Pre-clinical study in animal model: Cardio-metabolic effects of HIV protease inhibitors (Lopinavir/Ritonavir). Stellenbosch University, Stellenbosch, South Africa	Rats treated with either: mock surgery (sham), vehicle-, or protease inhibitor-containing pump for a total duration of 8 weeks	Increased LDL- and HDL-cholesterol levels and decreased cardiac contractile force with protease inhibitor treatment	Reyskens K. et al., PLoS One. 2013 ref. [19]
2. In vitro cell proliferation and cytotoxicity assays: HIV protease inhibitor ritonavir induces cytotoxicity of human endothelial cells. Emory University, Atlanta, USA	Human endothelial cells treated with ritonavir	Treatment with ritonavir resulted in significant endothelial cell injury	Zhong D. et al., *Arterioscler. Thromb. Vasc. Biol*. 2002 ref. [20]
3. Observational cohort study: Cardiovascular manifestations associated with interferon α2a Clinique Dermatologique, Hôtel-Dieu, Nantes, France	138 patients given interferon-α2a for various dermatological diseases between 1987 and 1990	Six cardiac manifestations were observed, one of which was a complete atrioventricular block	Mansat-Krzyzanowska E. et al., *Ann. Med. Interne*. 1991 ref. [75]

## Remdesivir

Remdesivir is an adenosine nucleotide prodrug. During replication of the viral genome, the remdesivir molecule is used by cell viral RNA-dependent RNA polymerase instead of adenosine which irreversibly interrupts viral replication [22]. Early compassionate use of remdesivir was provided to 53 patients with severe COVID-19 in the USA, Europe, Canada, and Japan during the very early onset of the pandemic (from January 25, 2020, through March 7, 2020) and observed clinical improvement in 68% of the patients [23]. A subsequent report of a double-blind, randomized, placebo-controlled trial in 1062 hospitalized patients with COVID-19 was published in November 2020, which demonstrated the benefits of remdesivir use, including the shortening of the time to recovery [24]. In another multicenter trial in Hubei, China (the initial epicenter of COVID-19), 237 patients with severe COVID-19 were enrolled and randomly assigned to either remdesivir group (*n* = 158) or placebo group (*n* = 79) between February 6 and March 12, 2020. Interestingly, remdesivir use was not associated with a difference in time to clinical improvement. However, the patients receiving remdesivir did have a faster time to clinical improvement than those receiving a placebo among the patients with symptom duration of 10 days or less [25]. These early studies had promoted the Emergency Use Authorization for remdesivir in the USA in May 2020.

On the other hand, in 2020, Gupta et al. evaluated two patients diagnosed with COVID-19 and received antibiotics (ceftriaxone and azithromycin), corticosteroids, and remdesivir. The first patient was a 26-year-old African American female with normal sinus rhythm with a heart rate of 80 to 100 bpm and a heart-rate corrected QT interval (QTc) of 439 ms. After her third dose of a five-day treatment course of remdesivir, she experienced sinus bradycardia (40–50 bmp), prolonged QTc interval of 555 ms, and T wave abnormality. The remdesivir treatment was discontinued, and the patient’s heart rate returned to baseline level with her QT interval stabilizing to 448 ms in three days. Notably, the co-administration of azithromycin could have contributed to the prolongation of the QT interval [26]. The second patient was a 77-year-old Caucasian female who was treated with antibiotics (ceftriaxone and azithromycin), steroid methylprednisolone, and remdesivir. On Day 3 after remdesivir administration, the patient developed severe sinus bradycardia with heart rate dropping from 80 bpm to 48 bpm. Consequently, remdesivir was discontinued, and the patient’s heart rate returned to baseline on subsequent days [26]. In November 2020, a 13-year-old boy with a history of episodic asthma was diagnosed with COVID-19 and started on therapy with remdesivir. After the third dose of remdesivir, the patient presented asymptomatic and non-hemodynamically significant sinus bradycardia (*i.e*. heart rate dropped from a baseline of 80–90 bpm to 40 bpm). After stopping remdesivir, his heart rate was normalized within 24 h. The authors postulated that bradycardia in this patient may be caused by remdesivir, which is an adenosine analog with adenosine-like blocking effects at the atrioventricular node [27].

Conversely, adenosine also acts as a potent vasodilator that can cause hypotension leading to the release of catecholamine, which may trigger ventricular tachycardia and ventricular fibrillation. It can also shorten atrial potential and refractoriness and lead to atrial fibrillation [22, 28]. Thus continuous cardiac rhythm monitoring is recommended in patients undergoing remdesivir treatment. It is especially advisable in individuals with pre-existing cardiac diseases or electrolyte disturbances [22]. Furthermore, it should also be noted that COVID-19 can have pathological effects on the cardiovascular system such as cardiac arrhythmia, heart failure, myocarditis, ischemic heart disease, and coagulation abnormality [29, 30]. Therefore, it can be challenging to differentiate between the multifactorial cardiovascular risks. It would be beneficial to perform detailed cardiovascular examinations before starting remdesivir for COVID-19 patients [29].

In 2021, Michaud et al. performed a study computing pharmacokinetic and pharmacodynamic data to measure affinity for blocking the rapid component of the delayed rectifier cardiac potassium current (*I*_Kr_) and the tendency toward a prolonged cardiac repolarization and torsade de pointes. The study revealed that remdesivir can significantly increase the risk of QT prolongation and torsade de pointes, especially with an elevated plasma concentration following intravenous administration [31].

## Paxlovid

Paxlovid is an oral treatment option for mild to moderate COVID-19 cases. It is co-packaged with two drugs, nirmatrelvir 150 mg and ritonavir 100 mg in its tablet form [32]. Pre-clinical safety studies with nirmatrelvir were essential in supporting the clinical development of Paxlovid. In a cardiovascular study by Sathish et al., oral nirmatrelvir was administered in telemetered monkeys. Nirmatrelvir at 75 mg/kg per dose produced a decrease in heart rate and increase in blood pressure, but nirmatrelvir at 20 mg/kg per dose did not produce such changes. There was also an increase in both the PR-interval and QT-interval, but these effects were considered to be secondary to the decrease in heart rate. There was a nirmatrelvir-related decrease in QTc. The repeat dose toxicity studies also showed no adverse findings for up to 1 month [33].

Since then, Paxlovid has been administered in high-risk COVID-19 patients with varied results. Ganipisetti et al. evaluated a 71-year-old woman who tested positive for COVID-19 and was started on a 5-day treatment course of Paxlovid. The patient subsequently developed syncopal episodes at home and was hospitalized for evaluation. She has no significant past medical history apart from the remote history of vasovagal syncopal episodes. She remained asymptomatic and appeared stable for discharge from the ER. While preparing for discharge, she suddenly developed lightheadedness, nausea with vomiting, and her heart rate suddenly dropped to 28 bpm, whereas her blood pressure was unrecordable. The patient was given a dose of 1 mg IV atropine push with which her heart rate was improved to 80 bpm instantly and her BP improved to 141/79 mmHg [32]. Upon literature search, there was no report of Paxlovid (nirmatrelvir-ritonavir) causing bradycardia/sinus dysfunction/syncope, especially when used alone. However, there were previous reports of bradycardia with ritonavir-based combinations such as lopinavir-ritonavir. It is possible that the ritonavir component has contributed to her presentation due to the fact that ritonavir is a strong CYP3A4 inhibitor and can increase the risk of cardiotoxicity when used concomitantly with certain medications. However, this patient was not on any medications other than Paxlovid. Awareness of possible side effects of bradycardia and sinus dysfunction with Paxlovid is essential until further studies to confirm the mechanism of action and frequency of occurance are available [32].

The use of Paxlovid has also been observed in the context of a heart transplant patient. The patient was a 28-year-old male who had been experiencing severe heart failure and underwent implantation of a total artificial heart as a bridge to transplantation. After the procedure, he contracted COVID-19 infection and was started on a course of nirmatrelvir/ritonavir along with a daily dose of dexamethasone. Patient oxygenation level was improved on the second day of therapy and the patient underwent successful heart transplantation after 71 days [34]. While this case study shows promising results for the administration of Paxlovid in patients requiring heart transplant, it is important to note the drug-drug interactions Paxlovid could potentially have with Tacrolimus. In a case study from May 2023, a 74-year-old male with history of heart transplantation and on maintenance immunosuppression with tacrolimus contracted COVID-19. He was prescribed antiviral therapy with Paxlovid. The patient complained of severe headaches, dehydration, and tremors. Laboratory investigation revealed a severely elevated tacrolimus level with acute renal injury. After the patient was taken off tacrolimus, his symptoms were improved [35]. The adverse effects associated with these drug-drug interactions, however, could potentially be managed with the use of phenytoin. In a similar case, a 67-year-old female with history of heart transplant received tacrolimus as part of her drug regimen. After being diagnosed with COVID-19, the patient was started on Paxlovid. Four days later, she presented to the emergency department for slowed speech, fatigue, weakness, and loss of appetite. Phenytoin was used and successfully decreased the patient’s tacrolimus level to within therapeutic range [36]. Another 43-year-old man with history of heart transplant due to viral myocarditis started taking Paxlovid after testing positive for COVID-19, and presented with worsening cough, dyspnea, and hemoptysis. After extensive work-up, tacrolimus toxicity was presumed to be the primary cause of illness. The patient was treated with phenytoin and his tacrolimus level was improved to 12.6 ng/mL [37]. For patients who have received a heart transplant, the use of a monoclonal antibody may also be preferred compared to Paxlovid. For example, a 76-year-old heart transplant recipient who was being maintained on tacrolimus contracted COVID-19 infection and was prescribed Paxlovid. His tacrolimus level was measured to be supratherapeutic at 49 ng/mL from 5.5 ng/mL in the month prior. After stopping Paxlovid, the patient received monoclonal antibody therapy with 175 mg of bebtelovimab and his tacrolimus level gradually decreased to an acceptable range (10.5 ng/mL) [38].

Additionally, increased risk of bleeding observed between Paxlovid and the drugs acting on the cardiovascular system such as ticagrelor, warfarin, and rivaroxaban. Dose adjustment and routine hematologic monitoring is recommended [39]. Rhabdomyolysis and myopathy are also potential interaction effects between lopinavir/ritonavir, Paxlovid, atazanavir, and atorvastin [39]. Given the drug-drug interactions involving Paxlovid, weighing the patient’s medication history before prescribing Paxlovid is highly important.

## Molnupiravir

In plasma, molnupiravir is converted to the active nucleoside analog (EIDD-1931) by host esterases. EIDD-1931 has been shown to inhibit a range of viruses including Chikungunya virus, Venezuelan equine encephalitis virus, RSV, norovirus, Influenza A and B viruses, Ebola virus, and human coronaviruses [40]. In a 2023 study, Tanbek et al. investigated the mechanisms of possible dose-dependent damage of molnupiravir on liver, lung, heart, and kidney tissues. Forty male rats were administered with molnupiravir (MOL) (10, 100, 1000 mg·kg^-1^·d^-1^) and total oxidant state (TOS) and oxidative stress index (OSI) levels were measured. The increase in TOS levels in the MOL10, MOL100, and MOL1000 groups was statistically significant compared to the control group. The increased OSI values in the MOL1000 group were significantly higher than the control and MOL10 groups. The study indicates that molnupiravir may cause tissue damage by disrupting the oxidant/antioxidant balance in different organs including the heart [41].

While it is advisable to be cautious when administering molnupiravir in COVID-19 patients, the drug has shown substantial benefits in lowering mortality and disease progression. A 2023 study comparing the effects between the use of molnupiravir and Paxlovid enrolled a total of 22098 patients with type 2 diabetes and confirmed SARS-CoV-2 infection. Most patients were older than 65 years, and molnupiravir users were older with more preexisting comorbidities. The results revealed that both molnupiravir and Paxlovid were associated with a lower risk of all-cause mortality and hospitalization in patients with COVID-19 and type 2 diabetes compared to non-use [42]. Similarly, the receipients of molnupiravir or Paxlovid was associated with significantly lower risks of all-cause mortality as compared with the non-receipients in a retrospective cohort study in 2022 [43]. In another multicenter prospective observational study, lower adverse events were reported for patients receiving molnupiravir than those receiving Paxlovid when observing individuals aged 65 and older. No significant difference was observed in individuals under 65 years old [44]. While more evidence is needed, molnupiravir could be considered as a potential treatment option for vaccinated older patients and high-risk patients. However, the use of molnupiravir could potentially be less beneficial in the context of lowering the risk of hospitalization. An observational study by Wong et al. found that, in patients infected with omicron variant of SARS-CoV-2, molnupiravir use was associated with lower risks of death and in-hospital disease progression than non-use, but risk of hospitalization was similar to the non-use group. However, Paxlovid users had lower risks of death, hospitalization, and in-hospital disease progression than non-users [45].

## Other repurposed antiviral drugs for treating COVID-19

### Hydroxychloroquine

Hydroxychloroquine (HCQ) is an antimalarial medication that has been repurposed for the treatment of COVID-19 as early as the first quarter of 2020 at the early onset of the pandemic. HCQ is a weak base that increases the pH of the intracellular vesicles like endosomes. These changes could affect several stages of viral life cycles from cell entry, viral replication, and viral particle assembly to viral particle release from the host cells [46]. Some clinical studies have found that after treatment with HCQ, the viral load significantly decreases or even disappears, and azithromycin can enhance the antiviral effect of HCQ [47, 48].

However, the use of HCQ may also have adverse cardiovascular side effects. In 2021, Tleyjah et al. conducted a meta-regression analysis on a total of 19 studies in 5652 patients. The pooled incidence of torsades de pointes arrhythmia, ventricular tachycardia, or cardiac arrest was 3 per 1000 in 18 studies with 3725 patients. Among 13 studies of 4334 patients, the pooled incidence of discontinuation of chloroquine (CQ) or HCQ due to prolonged QTc or arrhythmias was 5%. The pooled incidence of change in QTc from baseline of 60 ms to 500 ms or more was 9%. Mean age, coronary artery disease, hypertension, diabetes, concomitant QT-prolonging medications, intensive care unit admission, and severity of illness in the study populations explained between-studies heterogeneity [46].

There was a common practice to use HCQ in combination with azithromycin for COVID-19 during the pandemic. Azithromycin has been identified as a potential cause of serious cardiac arrhythmias through mechanisms dependent on and independent of QT prolongation and has been linked to an increased risk of sudden cardiac death [46, 49, 50]. Hence, the concomitant use of CQ or HCQ and azithromycin or other QT-prolonging agents could potentially increase the risk of serious cardiac arrhythmias and death, particularly in critically ill patients or those with risk factors for QT prolongation [46]. From January to June 2020, HCQ was one of the main suspected drugs for adverse drug reactions. Out of 272 cases with adverse effects, 84 cases were found when HCQ was used alone, and 151 cases were found when used in association with azithromycin [51]. Additionally, cardiac disorders were found in 58% of the cases related to HCQ, including 8 cases with fatal outcomes, and 13% of the cases related to lopinavir/ritonavir [51].

One possible mechanism for the adverse cardiovascular effects of HCQ may be due to the ability of HCQ to inhibit multiple K^+^ currents. First, CQ, HCQ and other quinines inhibit the cardiac *I*_K1_ current and can induce lethal ventricular arrhythmias [52]. CQ can bind to the cytoplasmic pore domain and block the cytoplasmic conduction pathway, which is stabilized by negatively charged and aromatic amino acids within a central pocket [53]. Alternatively, it can block K^+^ flow by interacting with negatively charged amino acids facing the ion permeation vestibule of the channels [54, 55]. CQ can also inhibit *I*_hERG_ which can lead to a prolongation of the QT interval [56]. It should be noted that inflammatory cytokines can also inhibit depolarizing K^+^ currents through downregulation of channel expression known as inflammatory channelopathy [57, 58]. Additionally, electrocardiogram changes including prolonged QT have been described in young athletes who suffer from long-COVID with prevalence as high as 27.5% [57, 59]. Therefore, more information is needed to determine whether these adverse effects are the result of the prescribed antivirals or COVID-19 infection.

These findings indicate that the cardiac risk imposed by CQ or HCQ use in COVID-19 disease is not trivial and there is a need for close monitoring of patients with COVID-19 who are treated with CQ or HCQ alone or in combination with azithromycin. Because their efficacy in improving the outcomes of patients with COVID-19 is lacking, these agents should be used only in the context of monitored clinical trials, given the potential harm that could be associated with their widespread use [46].

Furthermore, Nagaraja et al. studied the use of HCQ as prophylaxis in healthcare workers during the pandemic. Side effect profile analysis highlighted that 63 (37.9%) of participating healthcare professionals experienced at least one adverse drug reaction following the use of HCQ. Among them, gastrointestinal effects had the maximum incidence with 51 (30.7%) events of adverse drug reaction. This was followed by non-specific events 27 (16.2%), neurological effects 19 (11.4%), psychiatric 8 (4.8%), cardiovascular 6 (3.6%), dermatologic 6 (3.6%), ophthalmological 4 (2.4%) and respiratory 1 (0.6%). Cardiovascular symptoms included palpitations 6 (3.6%) and chest pain 2 (1.2%). Three of the four patients with palpitations experienced the symptoms with the first dose of the drug. Shortness of breath was experienced in 1 (0.6%) of subjects. Results also revealed that younger age (<40 years) (OR: 2.44, 95% CI: 1.18–5.05) was an independent risk factor for the development of side-effects [60]. In patients using HCQ as prophylaxis during the pandemic, proper surveillance is recommended especially after administration of the first dose and in patients of younger age.

### Ivermectin

Ivermectin was identified as an inhibitor of interactions between the human HIV integrase protein and the importin α/β 1 heterodimer. Notably, studies on SARS-CoV proteins have revealed a potential role of importin α/β 1 during the viral infection. Nuclear transport of viral proteins is essential for the replication cycle and inhibition of the host’s antiviral response [61–63].

Cardiovascular effects were first documented when ivermectin was used in the treatment of scabies. In a case report by Sparsa et al., an 86-year-old woman hospitalized for scabies was treated with benzyl benzoate and a single dose of ivermectin (200 µg/kg). She developed sinus tachycardia and asthma 3 days later. Screening for embolic, cardiac and infectious origins was found. Toxicity of ivermectin was suspected [64].

Since ivermectin has been repurposed as an antiviral agent and at higher doses than those for antiparasitic, the cardiovascular safety of ivermectin underwent further investigation. In isoflurane-anesthetized beagle dogs, ivermectin was administered in doses of 0.1 and 1 mg/kg and attained peak plasma concentrations of 0.94 ± 0.04 and 8.82 ± 1.25 μg/mL. Ivermectin decreased heart rate. However, there were no changes in mean blood pressure, suggesting that ivermectin does not cause hypotension or tachycardia directly. Ivermectin also prolonged QT interval/QTcV in a dose-dependent manner [65]. In previous studies, ivermectin directly inhibited *I*_Kr_ with IC_50_ of 12.52 μmol/L [66], and the human plasma protein binding ratio of ivermectin was reported to be 93.1% [67]. Therefore, the free peak concentration of ivermectin in the dog study is around 0.69 μmol/L, which is >18 times lower than the IC_50_ values for *I*_Kr_. These results, in addition to the lipid solubility of ivermectin, suggest that ivermectin could have accumulated in the heart [65]. Due to these cardiovascular safety profiles, patients with COVID-19 who are administered with ivermectin should be monitored closely for changes in heart rate or a prolonged QT interval.

## Antibody-based antivirals targeting SARS-CoV-2

Antibodies are a natural response for neutralizing viruses by either blocking the interaction between the virus and cell host or representing the viral antigens on the cell host to effector cells killing antibody-coated target cells. These cells permit mechanisms as Antibody-Dependent Cellular Cytotoxicity (ADCC), and Antibody-Dependent Cellular Phagocytosis (ADCP) or a way known as Complement-Dependent Cytotoxicity (CDC) to eliminate infected cells [68, 69]. Monoclonal antibodies (mAbs) owing to the unique properties of target specificity are an important class among therapeutic agents. mAbs neutralize viruses by blocking the interaction between the virus and host cell through opsonizing the free virus or binding to the required receptor on the cell surface for attaching and entering the virus into the cell [68–70].

The percentage of cardiovascular adverse events among total adverse events reported varied among the mAbs products. Sotrovimab had the lowest percentage of cardiovascular adverse events (5.7%) whereas tixagevimab + cilgavimab had the highest percentage of cardiovascular adverse events (13.0%). For all COVID-19 treatments, cardiac arrhythmia was the most frequently reported type of cardiovascular adverse event, accounting for 47% of cardiovascular adverse events reported or 4.7% of total adverse events. When examined by different types of mAbs products, hypertension was the most frequently reported type of cardiovascular adverse event for casirivimab + imdevimab, bamlanivimab, bamlanivimab + etesevimab, sotrovimab, and bebtelovimab, whereas embolic and thrombotic events were the most frequently reported type of cardiovascular adverse events for tocilizumab and tixagevimab + cilgavimab [71].

## Summary and perspectives

**Fig. 2:**
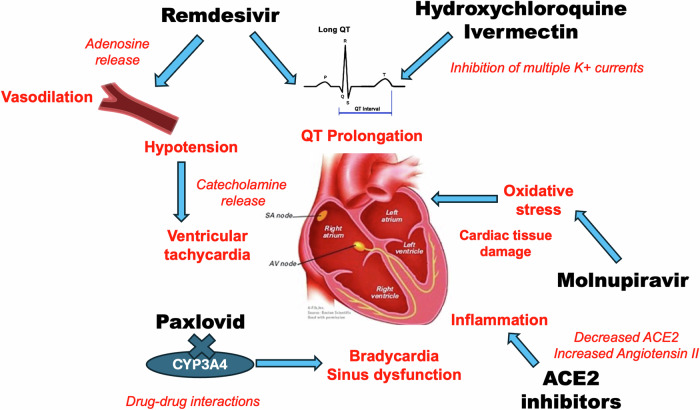
Illustrative summary of potential cardiovascular adverse effects of antiviral drugs for treating COVID-19. The highlighted key cardiovascular adverse effects of antivirals for treatment of COVID-19 include cardiac QT prolongation, ventricular tachycardia, bradycardia, sinus dysfunction, and cardiac tissue damage. The cellular mechanisms underlying these adverse effects may involve inhibition of K^+^ currents, drug-drug interactions via inhibition of cytochrome P450 3A4 (CYP3A4) by Paxlovid, release of adenosine or catecholamine, blockage of the atrioventricular node, and exacerbated oxidative stress in the heart. Specifically, co-administration of remdesivir and azithromycin is associated with QT prolongation. Hydroxychloroquine and ivermectin inhibit multiple K^+^ currents, also resulting in similar pro-arrhythmic effects. Use of remdesivir is also linked to ventricular tachycardia through the release of catecholamine in response to hypotension due to the drug-induced release of adenosine. Both remdesivir and Paxlovid are associated with bradycardia and sinus dysfunction. Molnupiravir disrupts oxidant/antioxidant balance in the heart which can result in cardiac tissue damage. Additionally, drugs that bind to angiotensin converting enzyme 2 (ACE2) may have adverse effects such as inflammation due to increased levels of Angiotensin II.

This review revisited the published evidence (Table 2) showing that remdesivir, Paxlovid, molnupiravir, HCQ, ivermectin, and other antibody-based antivirals are linked to adverse effects on the cardiovascular system (Fig. 2). Cardiovascular side effects of antiviral therapies may be a point of consideration in deciding whether it would be a good treatment option in patients diagnosed with COVID-19. Caution is advised when administering these antivirals, especially in patients who are at risk for cardiovascular events. Patients who are administered with these drugs should be closely monitored.

**Table 2:** Representative studies on cardiovascular complications of the antivirals targeting SARS- CoV-2 and COVID-19.

Study/Site Information	Subject Characteristics	Key Clinical Findings	Publications
1. Case study: Cardiac Adverse Events with Remdesivir in COVID-19 Infection. University of Miami Hospital, Miami, USA	2 patients with nasal polymerase chain reaction positive for SARS-CoV2	After treatment with Remdesivir, patients developed sinus bradycardia and drop in heart rate	Gupta A. et al., Cureus. 2020 ref. [26]
2. Pre-clinical safety study in animals: Comprehensive Nonclinical Safety Assessment of Nirmatrelvir Supporting Timely Development of the SARS-COV-2 Antiviral Therapeutic, Paxlovid. Pfizer Worldwide Research, Pfizer Inc, Pearl River, NY, USA	Nervous system/pulmonary study in rats and a cardiovascular study in telemetered monkeys	Increases in blood pressure and decreases in heart rate were observed only at the highest dose of Nirmatrelvir tested. No prolonged QTc-interval or arrhythmias observed.	Sathish J. et al., *Int. J. Toxicol*. 2022 ref. [33]
3. Case study: Paxlovid-Induced Symptomatic Bradycardia and Syncope. Presbyterian Hospital, Albuquerque, USA	Patient with multiple syncopal episodes who tested positive for COVID-19	Patient was started on a 5-day treatment course of Paxlovid and subsequently developed syncopal episodes at home and was hospitalized for evaluation	Ganipisette V. et al., *Cureus*. 2023 ref. [32]
4. Case study: Successful utilization of nirmatrelvir/ritonavir and dexamethasone in a patient with total artificial heart and COVID-19. King Saud Bin Abdulaziz University for Health Sciences, Saudi Arabia	28-year-old male with severe heart failure who underwent a procedure for implantation of a total artificial heart and contracted COVID-19 after the procedure	Patient oxygenation level was improved on the second day of therapy and the patient underwent successful heart transplantation after 71 days.	Alowais S. et al., *Medicine*. 2023 ref. [34]
5. Case study: Tacrolimus toxicity in the setting of concurrent Paxlovid use in a heart-transplant recipient. The Ross Heart Hospital at The Ohio State University Wexner Medical Center, Columbus, OH, USA	74-year-old male with history of heart transplantation and use of tacrolimus who contracted COVID-19	Patient complained of severe headaches, dehydration, and tremors after administration of Paxlovid. After patient was taken off tacrolimus, symptoms were improved.	Modi S. et al., *Eur. Heart J. Case Rep*. 2023 ref. [35]
6. Case study: Tacrolimus Drug-Drug Interaction with Nirmatrelvir/Ritonavir (Paxlovid™) Managed with Phenytoin. University of Iowa Hospitals and Clinics, Iowa City, USA	67-year-old female with history of heart transplant who received tacrolimus. She was started on Paxlovid after being diagnosed with COVID-19	Patient had a supratherapeutic tacrolimus level of 176.4 ng/mL. Tacrolimus levels were decreased after the administration of phenytoin.	Sindelar M. et al., *J Med Toxicol*. 2023 ref. [36]
7. Case study: Paxlovid with Caution: Novel Case of Paxlovid-Induced Tacrolimus Toxicity in a Cardiac Transplant Patient. Allegheny Health Network, Pittsburgh, PA, USA	43-year-old man with history of heart transplant who was started on Paxlovid after testing positive for COVID-19	Patient presented with worsening cough, dyspnea, and hemoptysis. Symptoms were presumed to be due to tacrolimus toxicity. Tacrolimus levels were improved after treatment with phenytoin.	Shah A. et al., *Eur. J. Case Rep. Intern. Med*. 2022 ref. [37]
8. Case study: Risks of paxlovid in a heart transplant recipient. Johns Hopkins School of Medicine, Baltimore, MD, USA	76-year-old heart transplant recipient maintained on tacrolimus who contracted COVID-19 infection	Patient’s tacrolimus level increased to supratherapeutic levels but gradually decreased after stopping Paxlovid and receiving monoclonal antibody therapy.	Stawiarski K. et al., *J. Heart Lung Transplant*. 2023 ref. [38]
9. Evidence-based review from six databases: Drug interaction risk between cardioprotective drugs and drugs used in treatment of COVID-19. SRM College of Pharmacy, Tamil Nadu, India.	Six databases: a) Micromedex drug interaction b) Medicine complete.com c) Liverpool Drug Interaction Group for COVID-19 therapies d) Epocrates e) Medscape f) drugs.com	Increased risk of bleeding was observed with concurrent use of Paxlovid and drugs acting on the cardiovascular system. Rhabdomyolysis, myopathy is also a potential interaction effect.	Shini Rubina S.K. et al., *Diabetes Metab. Syndr*. 2022 ref. [39]
10. Experimental animal study: Dose-dependent Oxidative Damage of Molnupiravir (Antiviral Drug for Treatment of COVID-19) in Lung, Liver, Heart, and Kidney Tissues in Rats. Faculty of Medicine, Inonu University, Malatya, Turkey	Fourty male Wistar albino rats administered with MOL (10-100-1000 mg/kg/day)	Oxidative stress is increased after administration of Molnupiravir in a dose-dependent manner.	Tanbek K. et al., *Arch Pharmacol Therapeut*. 2023 ref. [41]
11. Retrospective cohort study: Analysis of All-Cause Hospitalization and Death Among Nonhospitalized Patients With Type 2 Diabetes and SARS-CoV-2 Infection Treated With Molnupiravir or Nirmatrelvir-Ritonavir During the Omicron Wave in Hong Kong. Li Ka Shing Faculty of Medicine, The University of Hong Kong, Hong Kong, China	22,098 patients with type 2 diabetes and COVID-19	Treatment with molnupiravir or nirmatrelvir-ritonavir were both associated with a lower risk of all-cause mortality and hospitalization in patients with COVID-19 and type 2 diabetes.	Lui D.T.W. et al., *JAMA Netw Open*. 2023 ref. [42]
12. Retrospective cohort study: Real-world effectiveness of early molnupiravir or nirmatrelvir-ritonavir in hospitalized patients with COVID-19 without supplemental oxygen requirement on admission during Hong Kong’s omicron BA.2 wave. Department of Pharmacology and Pharmacy, LKS Faculty of Medicine, University of Hong Kong, Hong Kong, China	Patients in Hong Kong who were hospitalized with a confirmed diagnosis of SARS-CoV-2 infection between Feb 26 and April 26, 2022.	Risks of all-cause mortality were lower with receipt of both molnupiravir or nirmatrelvir/ritonavir compared with non-receipt.	Wong C.K.H. et al., *Lancet Infect. Dis*. 2022 ref. [43]
13. Multicenter prospective observational study: Effectiveness and Adverse Events of Nirmatrelvir/Ritonavir Versus Molnupiravir for COVID-19 in Outpatient Setting. Kangnam Sacred Heart Hospital, Hallym University College of Medicine, Seoul, Korea	Patients aged 18 years and older who were diagnosed with COVID-19	Patients 65 and older who were vaccinated had a reduced risk of adverse events after receiving molnupiravir. For patients in this age group, only nirmatrelvir/ritonavir was significantly associated with adverse effects.	Park J.J. et al., *J. Korean Med. Sci*. 2023 ref. [44]
14. Observational study: Real-world effectiveness of molnupiravir and nirmatrelvir plus ritonavir against mortality, hospitalization, and in-hospital outcomes among community-dwelling, ambulatory patients with confirmed SARS-CoV-2 infection during the omicron wave in Hong Kong. LKS Faculty of Medicine, University of Hong Kong, China	Patients with COVID-19 who received either molnupiravir (800 mg twice daily for 5 days) or nirmatrelvir plus ritonavir (nirmatrelvir 300 mg and ritonavir 100 mg twice daily for 5 days, or nirmatrelvir 150 mg and ritonavir 100 mg if estimated glomerular filtration rate was 30-59 mL/min per 1.73 m^2^)	Both molnupiravir and nirmatrelvir/ritonavir use was associated with lower risks of death and in-hospital disease progression than non-use. Nirmatrelvir/ritonavir use was also associated with lower risks of hospitalization.	Wong C.K.H et al., *Lancet*. 2022 ref. [45]
15. Meta-analysis of 19 single-center and multicenter studies: Cardiac Toxicity of Chloroquine or Hydroxychloroquine in Patients with COVID-19: A Systematic Review and Meta-regression Analysis. King Fahad Medical City, Riyadh, Saudi Arabia	Total of 5652 patients with COVID-19 treated with CQ or HCQ, with or without azithromycin	Treatment of patients with COVID-19 with CQ or HCQ is associated with an important risk of drug-induced QT prolongation and relatively higher incidence of torsades de pointes, ventricular tachycardia, or cardiac arrest	Tleyjeh I. et al., *Mayo Clin. Proc. Innov. Qual. Outcomes*. 2021 ref. [46]
16. Descriptive study: Pharmacovigilance follow-up of patients in the context of the COVID-19 pandemic. Centre régional de pharmacovigilance de Bourgogne, University Hospital, Dijon, France	2189 included cases, 67.1% were serious. Cases were mainly related to other drugs administered to COVID-19 positive patients (58.5%) followed by drugs used to treat COVID-19 (33.7%) and drugs suspected of having promoted COVID-19 (7.8%)	Hydroxychloroquine was the main suspected drugs for adverse drug reactions. Cardiac disorders were found in 58% of cases related to hydroxychloroquine.	Grandvuillemin A. et al., *Therapie*. 2023 ref. [51]
17. Retrospective, cross-sectional study: HyPE study: hydroxychloroquine prophylaxis-related adverse events’ analysis among healthcare workers during COVID-19 pandemic: a rising public health concern. Bowring and Lady Curzon Hospital, Bangalore, India	COVID-19 negative and asymptomatic healthcare workers, taking hydroxychloroquine prophylaxis	A higher incidence of adverse events was observed after first dose of hydroxychloroquine of which cardiovascular effects were 3.6%	Nagaraja B. et al., *J. Public Health*. 2020 ref. [60]
18. Case report: Systemic adverse reactions with ivermectin treatment of scabies. Service de Dermatologie, CHU Dupuytren, Limoges Cedex, France	86-year-old diagnosed with and treated with Ivermectin	The patient developed sinus tachycardia and asthma 3 days later.	Sparsa A. et al., *Ann. Dermatol. Venereol*. 2006 ref. [64]
19. Pre-clinical study in animals: Cardiovascular safety pharmacology of ivermectin assessed using the isoflurane-anesthetized beagle dogs. Department of Pharmacology, Faculty of Medicine, Toho University, Tokyo, Japan	Isoflurane-anesthetized beagle dogs (*n* = 4). Ivermectin in doses of 0.1 followed by 1 mg/kg	Ivermectin decreased heart rate without altering mean blood pressure, and prolonged QT interval/QTcV in a dose-related manner	Suzuki N. et al., *J. Toxicol. Sci*. 2023 ref. [65]
20. Disproportionality analyses: Cardiovascular Adverse Events Associated with Monoclonal Antibody Products in Patients with COVID-19. Tongji Hospital, Huazhong University of Science and Technology, Wuhan, China	Patient with COVID-19 with use of seven monoclonal antibody products (casirivimab + imdevimab, bamlanivimab, bamlanivimab + etesevimab, sotrovimab, tocilizumab, bebtelovimab, tixagevimab + cilgavimab)	The percentage of cardiovascular adverse events among total adverse events reported varied among monoclonal antibody products	Zou J. et al., *Pharmaceuticals*. 2022 ref. [71]

There are a number of remaining dilemmas, such as whether to increase ACE2 levels in tissues to inhibit the inflammatory response or promote the reduction of tissue ACE2 levels to decrease viral entry and replication of SARS-CoV-2 [72]. Interestingly, Oliveira et al. performed molecular docking with the ACE2 receptor in complex with S-glycoprotein of SARS-CoV-2 using 60 candidate drugs. The docking result showed that paritaprevir and ivermectin have the highest binding affinity to the ACE2 receptor. Remdesivir and azithromycin are in the second group of promising drugs [73], the promising binding affinities of these drugs to ACE2 highlight their potential as treatments for COVID-19. However, the potential disruption of ACE2 function could also lead to implications beyond their antiviral effects, such as inflammation (Fig. 2). In addition, it is still uncertain about how to practically differentiate the cardiovascular injuries caused by SARS-CoV-2 virus itself (in both acute and long-COVID phases) with the cardiovascular adversity triggered by some antiviral therapies commonly used during the COVID-19 pandemic. Further studies are needed to identify the main pathogenic drivers and specific biomarkers for improving the differential diagnosis and benefit-risk balanced management strategy of cardiovascular problems caused by either the viruses or the antiviral drugs.

One of the major challenges thus far has been the ability to predict the adverse effects of antivirals used in COVID-19 treatment. More accurate predictions could potentially be made with the development of the newest AlphaFold 3 artificial intelligence (AI) platform, which is able to accurately predict the structure of a wide range of biomolecular systems such as proteins, nucleic acids, small molecules and ions in a unified framework [74]. Since the structures of biomolecules play a crucial role in determining their cytotoxicity, the AlphaFold 3 AI platform can hold immense potential in identifying the possible key sites within antivirals that may cause cardiovascular side-effects and in determining which modifications of the biomolecules might reverse the adverse effects. Furthermore, understanding the structure of biomolecules may also assist researchers to analyze drugs interactions with their target molecules and to discover new antivirals. With the introduction of AlphaFold 3, we anticipate considerable advancements in the development of novel and better COVID-19 drugs in the foreseeable future.
